# Stabilization of negative activation voltages of Cav1.3 L-Type Ca^2+^-channels by alternative splicing

**DOI:** 10.1080/19336950.2020.1859260

**Published:** 2020-12-31

**Authors:** Nadja T. Hofer, Alexandra Pinggera, Yuliia V. Nikonishyna, Petronel Tuluc, Eva M. Fritz, Gerald J. Obermair, Jörg Striessnig

**Affiliations:** a Department of Pharmacology and Toxicology, Centre for Molecular Biosciences, University of Innsbruck, Austria; b Neurobiology Division, MRC Laboratory of Molecular Biology, Cambridge, UK; c Institute of Physiology, Medical University Innsbruck, Innsbruck, Austria; d Division Physiology, Karl Landsteiner University of Health Sciences, Krems, Austria

**Keywords:** Cav1.3, Ca^2+^ channels, L-type, alternative splicing, disease genetics

## Abstract

-->Low voltage-activated Cav1.3 L-type Ca^2+^-channels are key regulators of neuronal excitability controlling neuronal development and different types of learning and memory. Their physiological functions are enabled by their negative activation voltage-range, which allows Cav1.3 to be active at subthreshold voltages. Alternative splicing in the C-terminus of their pore-forming α1-subunits gives rise to C-terminal long (Cav1.3_L_) and short (Cav1.3_S_) splice variants allowing Cav1.3_S_ to activate at even more negative voltages than Cav1.3_L_. We discovered that inclusion of exons 8b, 11, and 32 in Cav1.3_S_ further shifts activation (-3 to -4 mV) and inactivation (-4 to -6 mV) to more negative voltages as revealed by functional characterization in tsA-201 cells. We found transcripts of these exons in mouse chromaffin cells, the cochlea, and the brain. Our data further suggest that Cav1.3-containing exons 11 and 32 constitute a significant part of native channels in the brain. We therefore investigated the effect of these splice variants on human disease variants. Splicing did not prevent the gating defects of the previously reported human pathogenic variant S652L, which further shifted the voltage-dependence of activation of exon 11-containing channels by more than -12 mV. In contrast, we found no evidence for gating changes of the *CACNA1D* missense variant R498L, located in exon 11, which has recently been identified in a patient with an epileptic syndrome.

Our data demonstrate that alternative splicing outside the C-terminus involving exons 11 and 32 contributes to channel fine-tuning by stabilizing negative activation and inactivation gating properties of wild-type and mutant Cav1.3 channels.

## Introduction

In electrically excitable cells voltage-gated Ca^2+^-channels (VGCCs) are key regulators of plasmalemmal Ca^2+^-entry in response to changes in membrane potentials. Ca^2+^-influx promotes membrane depolarization and action potential propagation and serves as an important intracellular second messenger. The resulting Ca^2+^-transients regulate Ca^2+^-dependent physiological functions such as hormone secretion, muscle contraction, neurotransmitter release and gene transcription associated with synaptic plasticity, learning, and memory [1,2].

Within the class of L-type Ca^2+^-channels (LTCC; Cav1.1–1.4 [3]) Cav1.3 channels activate at more negative potentials [4], which is essential for supporting specialized physiological functions such as pacemaking activity in the sinoatrial node (SAN) [5] as well as in adrenal chromaffin cells [6] and hearing function in cochlear inner hair cells (IHCs) [7,8]. In addition, this allows them to serve as key regulators of neuronal excitability and to stabilize plateau potentials in neurons [9]. Cav1.3 channels also inactivate at more negative voltages than other LTCCs. This can reduce their availability in cells with more positive resting membrane potentials, thus preventing Ca^2+^-toxicity.

The diverse physiological functions of Cav1.3 channels, ranging from tonic neurotransmitter release in IHCs to pacemaking in the SAN [10] and dopamine neurons [11,12], require the cell-specific fine-tuning of its gating properties. One such mechanism is alternative splicing. We and others have shown [13–15] that alternative splicing in the C-terminus produces strong effects on channel gating. Cav1.3 α1-subunit splice variants with a short C-terminus (Cav1.3_S_) lacking a C-terminal modulatory domain further shift the activation voltage range by >7 mV to even more negative voltages [15]. Since short and long splice variants are expressed at comparable levels in many brain regions [15] and even in individual neurons [16], this indicates that fine-tuning by alternative splicing is required for proper cellular function. This is supported by the observation that elimination of splicing-induced differences in the C-terminal modulatory domain in mice alters cell excitability (as shown in chromaffin cells of mutant Cav1.3DCRD^HA/HA^ mice [17]). Moreover, human *de novo* missense variants, located at different regions of the channel, causing high risk for severe neurodevelopmental disorders [18,19] also shift the voltage-dependence of channel gating to more negative voltages (such as recently shown for mutation S652L [20]).

This raises the important question if alternative splicing outside the Cav1.3 α1-subunit C-terminus is also involved in fine-tuning channel activity and if this also persists in disease variants. Whereas C-terminal splicing is well characterized, the contribution of alternative splicing to channel gating in other channel domains is largely unexplored. We took advantage of the unexpected observation that Cav1.3 channel activation in a previously characterized stable cell line [16] occurs ~-6 mV more negative than in transiently expressed channels. The Cav1.3 α1-splice variant used for generating the stable cells differs from the transiently expressed α1-subunit construct (Genbank accession number EU363339; referred to as Cav1.3_8a-42_ or Cav1.3 _L_ in this report) by containing exon 8b instead of exon 8a plus additional exons 11 and 32 and, in case of the C-terminally long isoform, an additional exon 44. This prompted us to hypothesize that alternative exon 8b and/or additional exons 11 and 32 might be responsible for this negative shift. Exons 8a/b are mutually exclusive exons located in the first domain in segment 6 (IS6) and are essential for building a functional pore. However, the functional significance of exon 8 splicing remains unknown [10]. Only few studies systematically analyzed the effect of alternative exon 11 and exon 32 splicing in Cav1.3 α1-subunits on channel gating [21,22] but results are inconclusive and the expression pattern of these exons in different tissues remains elusive.

We therefore functionally characterized various Cav1.3_S_ splice variants containing either exon 8a or 8b with or without exons 11 and 32 or a combination of both. In accordance with our hypothesis, we provide evidence that all combinations can stabilize more negative activation (-3 to -4 mV) and inactivation voltages (-4 to -6 mV) of Cav1.3_S_. Moreover, we show that this negative shift of activation voltage adds to the negative shift induced by pathogenic *CACNA1D* mutations [19,23] as demonstrated for mutation S652L. This newly identified role of exon 11 also prompted us to study the pathogenic potential of a *CACNA1D* missense mutation in exon 11, R498L, recently identified in a patient with an epileptic syndrome [24]. The absence of any measurable phenotype makes a pathogenic role of this mutation unlikely.

## Materials and Methods

### cDNA constructs

Human Cav1.3 constructs were used. Construct Cav1.3 _L_ (here termed Cav1.3_8a-42)_) contains exons 8a and 42 and lacks exons 11 and 32). It corresponds to Genbank accession number EU363339. The corresponding C-terminally short variant Cav1.3_8a-43s_, previously also termed Cav1.3_43s_, is the corresponding C-terminally short variant containing exons 8a and 43S [15].

*Cav1.3_8b-43S_*: Exon 8b was inserted from Cav1.3_8b-42_^4^ into Cav1.3_8a-43S_^15^ using AgeI and SalI restriction sites.

*Cav1.3_8a/b-11-43S_* and *Cav1.3_8a/b-32-43S_*: Constructs have been generated utilizing splicing by overlap extension (SOE) PCR to splice additional exon 11 or exon 32 into Cav1.3_8a/b-43S_ constructs. Briefly, in the 1st step PCR desired fragments were amplified in two separate PCRs (PCR a and b) using overlapping primers (primer pair 1 and 2 for Cav1.3_8a/b-11-43S_ or primer pair 4 and 5 for Cav1.3_8a/b-32-43S_) containing the sequence of exon 11 or exon 32 at the 5´-end as an overhang. Cav1.3_8a-43S_ for constructs containing exon 8a or Cav1.3_8b-43S_ for constructs containing exon 8b were used as a template. In the 2nd step PCR (PCR c) both PCR products were used as templates and were combined with flanking primers (primer pair 3 for Cav1.3_8a/b-11-43S_ or primer pair 6 for Cav1.3_8a/8b-32-43S_). For primer sequences and PCR reaction mix see Suppl. Methods.

*Cav1.3_8a/b-11-32-43S_* was generated from Cav1.3_8a/b-11-43S_ and Cav1.3_8a/b-32-43S_ using BglII to HindIII restriction sites.

*S652L_8b-11-43S_, R498L_8a-11-42_*: Respective mutations were introduced into C-terminal short Cav1.3 splice variant containing exon 8b, 11, and 43S (Cav1.3_8b-11-43S_) or long splice variants (Cav1.3_8a-11-42_ or Cav1.3_8a-42_) using SOE PCR as described above. Briefly, corresponding Cav1.3 loci were amplified using overlapping primers to introduce the respective mutations in two separate PCR reactions. Resulting products were combined in a final PCR reaction and inserted into respective Cav1.3_8b-11-43S_, Cav1.3_8a-11-42_ or Cav1.3_8a-42_ sites using restriction digestion. For primer sequences and PCR reaction mix see Supplemental Methods.

### Cell culture and transfection

For whole-cell patch-clamp recordings, tsA-201-cells (a human embryonic kidney (HEK)-293 subclone stably expressing SV40 temperature-sensitive T-antigen; European Collection of Authenticated Cell Cultures, ECACC, 96,121,229) or HEK-293 cells stably expressing β_3_ and α_2_δ -1 were cultured in Dulbecco´s modified Eagle´s medium (DMEM; Cat# D6546; Merck KGaA, Darmstadt, Germany) containing 4500 mg/l L-glucose, 10% fetal bovine serum (FBS; Cat# 10,270,106; Thermo Fisher Scientific, Waltham, MA, USA), 2 mM L-glutamine (Cat# 25,030,032; Thermo Fisher Scientific, Waltham, MA, USA), 10 units/ml penicillin G (Cat# P-3032; Merck KGaA, Darmstadt, Germany), 10 µg/ml streptomycin (Cat# S-6501; Merck KGaA, Darmstadt, Germany) and maintained at 37°C in a humidified incubator with 5% CO_2_. Cells were grown to ~80% confluency and split using 0.05% trypsin for cell dissociation. HEK-293 cells were periodically treated with selection agents for each subunit (β_3_, 500 µg/ml geneticin (Cat# 10,131,027; Thermo Fisher Scientific, Waltham, MA, USA); ɑ_2_δ-1, 10 µg/ml blasticidin S HCl (Cat# A1113903; Thermo Fisher Scientific, Waltham, MA, USA)). For recordings of all Cav1.3 8a and 8b splice variants and wild-type Cav1.3_8b-11-43S_ vs S652L_8b-11-43S_, HEK-293 cells stably expressing human β_3_ and ɑ_2_δ-1 were transiently transfected with desired LTCC ɑ_1_ (3 µg) using the Ca^2+^-phosphate precipitation method [25]. For recordings of Cav1.3_8a-42,_ Cav1.3_8a-11-42_ vs R498L_8a-11-42_ tsA-201 cells were transiently transfected with human ɑ_1_ (3 μg), rat β_3_ (2 μg; Genbank accession number NM_012828), and rabbit ɑ_2_δ-1 (2.5 μg, Genbank accession number NM_001082276) subunits [15,16,26]. Co-transfected EGFP (1.5 µg) served as a transfection marker. All data were obtained from at least three independent transfections. On the following day, cells were trypsinized (0.05% trypsin) and plated onto poly-L-lysine-(Cat# P-2636; Merck KGaA, Darmstadt, Germany) precoated 35 mm culture dishes. Cells were kept at 30°C and 5% CO_2_ and were subjected to electrophysiological experiments 20–72 h after transfection.

### Electrophysiological recordings in HEK-293 cells

For whole-cell patch-clamp experiments, patch pipettes were pulled in a micropipette puller (Sutter Instrument, Novato, CA, USA) using borosilicate glass capillaries (borosilicate glass; Cat# 64–0792, Warner Instruments, Hamden, CT, USA) and fire-polished using a MF-830 microforge (Narishige Co, Tokyo, Japan). Pipettes with a resistance of 1.5 - 3 MΩ were backfilled with internal solution containing (in mM): 135 CsCl, 10 Cs-EGTA, 1 MgCl_2_, 10 HEPES, 4 ATP-Na_2_ adjusted to pH 7.4 with CsOH. The bath solution contained (in mM): 15 CaCl_2_, 150 Choline-Cl, 1 MgCl_2_, 10 HEPES, adjusted to pH 7.3 with CsOH. Whole-cell patch-clamp recordings were performed at room temperature (20–23°C) using an Axopatch 200B Amplifier (Molecular Devices, San José, CA, USA). Data were digitized (Digidata, 1322A digitizer, Molecular Devices, San José, CA, USA) at 50 kHz, low-pass filtered at 1–5 kHz and analyzed using pClamp 10.2 software (Molecular Devices, San José, CA, USA). Series resistance was compensated by 60–90% and all voltages were corrected for a liquid junction potential of -9.3 mV [26]. Currents were leak subtracted either offline using a 50 ms hyperpolarizing voltage step from -80 to -90 mV or using an online P/4 protocol. Current-voltage (I–V) relationships were measured by applying 20 or 50 ms depolarizing square pulses to various test potentials (Δ 5 mV increments) starting from a holding potential (HP) of -89 mV. I–V curves where fit to the equation I = G_max_ (V–V_rev_)/(1 + exp[- (V–V_0.5_)/k]) where I is the peak current, G_max_ is the maximum conductance, V is the test potential, V_rev_ is the extrapolated reversal potential, V_0.5_ is the half-maximal activation voltage, and k is the slope factor. The voltage dependence of activation was obtained from the I–V relationship by calculating the conductance (G = I/V–V_rev_) followed by normalization (G/G_max_) and plotting as a function of voltage. The G-V curve was fit using following Boltzmann relationship: G = G_max_/(1 + exp[- (V–V_0.5_)/k]. The steady-state inactivation was determined by calculating the ratio between current amplitudes of a control versus a test pulse (I/I_control_; both 20 ms to V_max_) separated by a 5-s conditioning step to various potentials (Δ 10 mV increments; 30 s intersweep interval; HP, -89 mV) and plotting as a function of voltage. Steady-state inactivation curves were fit using a modified Boltzmann equation: I(V) = (1 – I_max_)/(1 + exp[(V–V_0.5,inact_)/k_inact_] + I_max_ where V_0.5inact_ is the half-maximal inactivation voltage and k_inact_ is the inactivation slope factor. The percentage of inactivation during a 5-s long depolarizing pulse from a HP of -89 mV to the potential of maximal inward current (V_max_) was determined after 50, 100, 250, 500, 1000, and 5000 ms with Ca^2+^ as charge carrier. In general, experiments with currents <100 pA and >1000 pA were prospectively excluded from analysis.

### RNA isolation and reverse transcription

All tissues were obtained from male C57BL6/N mice. Mice for expression profiling were taken at 8–12 weeks for brain preparations, ∼4 months for mouse chromaffin cell (MCC) preparation and postnatal day (P) 23 for cochlea preparations (after hearing onset). For qRT-PCR, whole brains were prepared from 3 or 12 weeks old animals and cortex, cerebellum, hippocampus, striatum, ventral tegmental area (VTA), and substantia nigra (SN) samples were dissected from brains of 12–14 weeks old mice. For whole brains, hippocampus, and striatum samples, only one hemisphere was processed for analysis. Mice were anesthetized with isoflurane (Vetflurane, Vibac UK, 1000 mg/g) and sacrificed by cervical dislocation. Brain samples were quickly removed, snap frozen in liquid nitrogen and stored at -80°C. Cochlea, MCCs, and brain regions were dissected in ice-cold 1x PBS buffer using a dissecting microscope. For MCC preparations, cortical tissue was removed and medullae were snap frozen in liquid nitrogen. For cochlea preparations, the bony cochlear capsule was carefully removed and the extracted cochlear helix was immediately snap frozen in liquid nitrogen.

To dissect VTA and SN freshly extracted brains were snap frozen in isopentane pre-cooled with dry ice (∼-40°C). 100 µm thick sections were cut on a cryostat (CM1950, Leica, Germany) and collected on glass coverslips. The sections were immediately re-frozen on dry-ice and punched under a dissection microscope using a pre-cooled sample corer (VTA: inner diameter 0.8 mm, 1 punch per hemisphere; SN: inner diameter 0.5 mm, 2 punches per hemisphere). For each brain region, tissue punches from both hemispheres of 7–8 successive 100 µm sections between Bregma -3.00 mm to -3.80 mm (according to Paxinos [27]) were collected in the sample corer (Fine Science Tool, Germany). The tissue punches were transferred into an Eppendorf tube and again snap frozen in liquid nitrogen. The punched sections were stained with cresyl violet for histological verification.

Purification of total RNA from all samples was implemented using Qiagen RNeasy lipid tissue mini kit (Qiagen, GmbH, Hilden, Germany) according to the manufacturer’s manual. On the day of preparation individual samples were placed in a round-bottom tube and were lysed with an appropriate amount of phenol/guanidine-based Qiazol lysis reagent. The tissue was then homogenized using a rotor-stator homogenizer. For very small amounts of tissue, homogenization was performed by passing the lysate through a 21-gauge needle. An optional on-column DNase digestion was performed to reduce genomic DNA contamination. Samples were eluted with 2 × 15 or 30 µl nuclease-free water. The RNA concentration was determined photometrically yielding approximately 20 ng/µl for VTA and SN, 60–100 ng/µl for cochlea and MCCs, 300–800 ng/µl for brain regions and 1–2 µg/µl RNA for whole-brain samples with high purity. 1 µg of total RNA was reverse transcribed using Maxima H Minus First Strand cDNA synthesis kit with random hexamer primers (Thermo Fisher Scientific, Waltham, MA, USA). 1 µl cDNA corresponds to 50 ng RNA equivalent. In case of low RNA yield, 13 µl of total RNA was reverse transcribed. Therefore, 1 µl cDNA corresponds to 0.65x the amount of RNA equivalent.

Expression profiling of Cav1.3 splice variants by nested PCR and quantitative real-time PCR using a standard curve-based approach [28] is described in detail in the supplemental methods.

### Statistics

Data were analyzed using Clampfit 10.2 (Axon Instruments) and Sigma Plot 11 (Systat Software, Chicago, IL). For statistical analysis Graph Pad Prism 5.01 software (GraphPad Software, La Jolla, CA) was used. Significance of differences between two groups was determined using unpaired student´s t-test for normally distributed data. Significance between three and more groups was determined using one-way analysis of variance (ANOVA) for normally distributed data (with Bonferroni posttest as indicated). All data are represented as mean ± SEM. Significance level was set to α-error lower than p < 0.05 (*), p < 0.01 (**) and p < 0.001 (***).

## Results

### Alternative splicing of Cav1.3 α1-subunits in exons 8, 11, and 32

**Figure 1.**
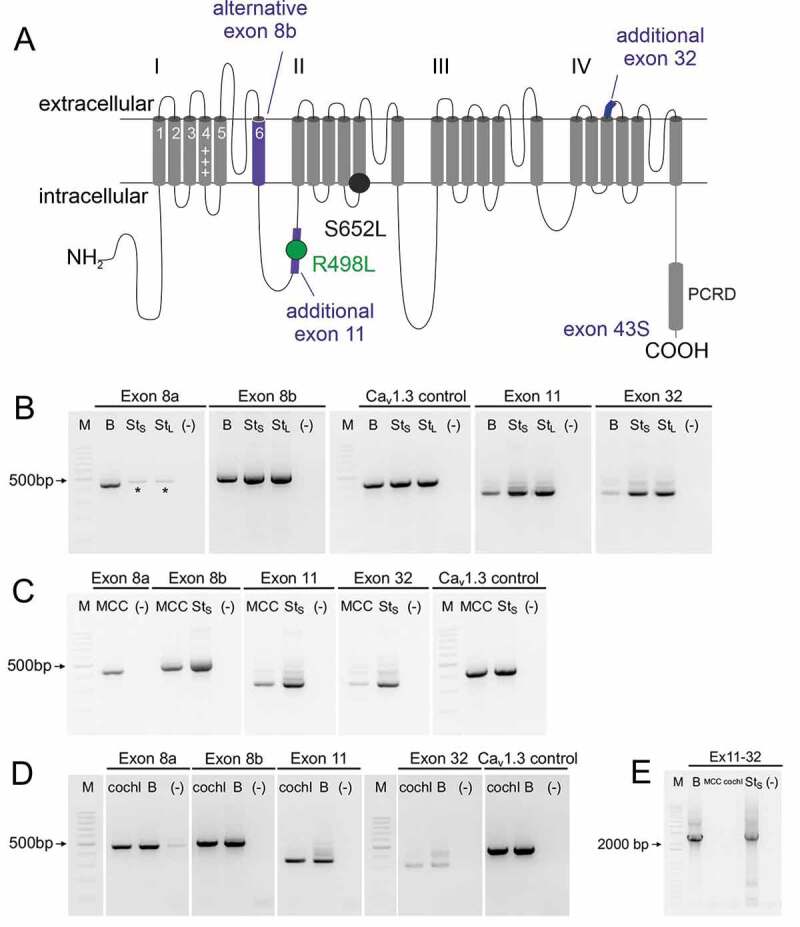
Expression profiling of Cav1.3 splice variants in brain, chromaffin cells and cochlea

To test the hypothesis that alternative splicing outside the C-terminus can further shift voltage-dependent gating properties of short Cav1.3_S_ variants (here we employed the short variant produced by exon 43S splicing (Cav1.3_43S_ [15]), we first performed nested PCR to investigate the expression of exons 8a/b, 11 and 32 in tissues with high abundance of Cav1.3 channels. Exons 8a, 8b, 11, and 32 (Figure 1a, Fig. S1) were detected in samples of mouse whole brain (B; Figure 1b), mouse chromaffin cells (MCC; Figure 1c) and in cochlea preparations (cochl; Figure 1d) (confirmed by sequencing). Furthermore, by employing long-range PCR we could show that exons 11 and 32 are expressed together within single transcripts in the brain. These were not detected in MCCs or cochlea preparations (Figure 1e).

A. Transmembrane topology of the Cav1.3 α1-subunit with a short C-terminus (due to usage of exon 43S). Spliced exons are indicated in blue. The positions of *de novo* missense mutations are also indicated (circles).

B-D. Detection of exons 8a, 8b, 11, and 32 in brain (B), mouse chromaffin cells (C) and cochlea (D). E. Detection of transcripts containing exons 11 and 32 in the brain but not in mouse chromaffin cells and the cochlea. One representative gel out of 2–5 nested PCR experiments per tissue sample is illustrated. 50–100 ng RNA equivalent for outer PCR and 0.5–1 µl of 1^st^ PCR product for inner PCR were used. Fragments were generated by specific primers for exon 8a (439 bp), exon 8b (471 bp), exon 11 (305 bp), exon 32 (292 bp), Cav1.3 control (426 bp) and exon 11–32 (2483 bp), respectively. *, unspecific band for St_S_ and St_L_ likely representing amplification of exon 8b with exon 8a primers due to high sequence similarity. B, whole brain without cerebellum; MCC, mouse chromaffin cells; cochl, whole cochlea; St_S_, positive control, stable cell line expressing Cav1.3_S_ together with exons 8b, 11, and 32; St_L_, positive control, stable cell line expressing Cav1.3 _L_ together with exons 8b, 11 and 32; (-): negative control, primers against mouse or human sequence, no template.

**Figure 2.**
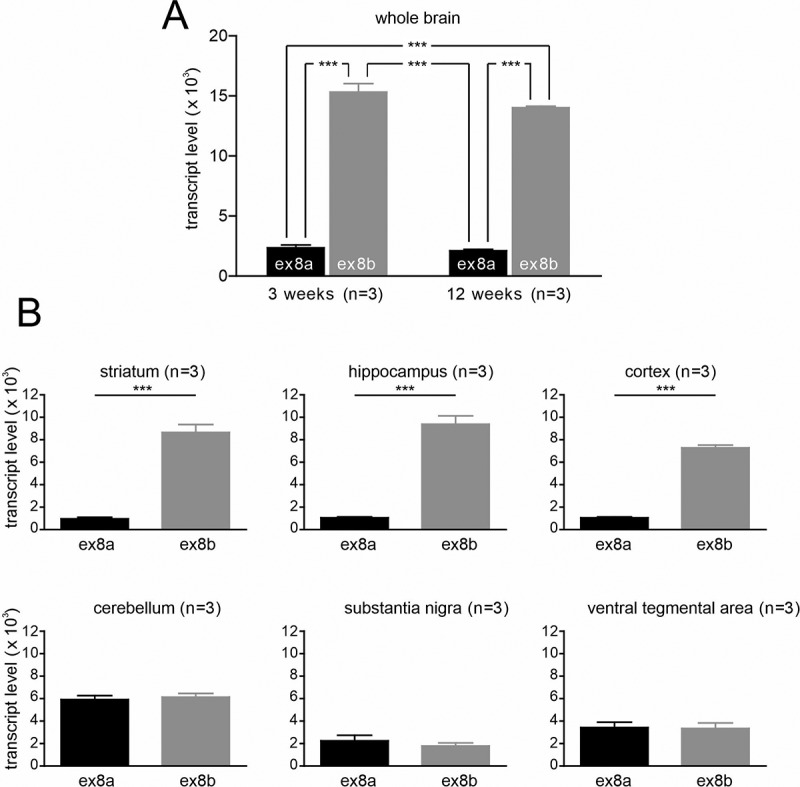
Cav1.3 exon 8a and 8b expression in mouse brain preparations.

Since disease-causing *de novo* missense variants have been found in both exon 8a [29,30] and exon 8b [31,32] we therefore also determined which of the two variants is the predominant form in the brain (Figure 2). We quantified their expression using standard curve-based absolute qRT-PCR (for details and specificity of the custom-designed assays see also Methods and Fig. S2). Expression of exon 8b was approximately sixfold higher in whole brain samples compared to exon 8a (Figure 2a). There was no difference in the expression pattern of exon 8a and 8b between 3- and 12-week-old mice. Brain region-specific analysis revealed significantly higher expression of exon 8b in the cortex (∼sevenfold), hippocampus, and the striatum (both ∼ninefold) compared to exon 8a whereas the expression levels of exon 8a and 8b were similar in the cerebellum, substantia nigra (SN) and ventral tegmental area (VTA) (Figure 2b).

A. Expression levels of Cav1.3 exon 8a and 8b in whole brain samples of 3 weeks (n = 3) or 12 weeks (n = 3) old male C57BL6/N mice using quantitative real-time PCR.

B. Expression levels of Cav1.3 exon 8a and 8b in indicated brain regions of 12–14 weeks old C57BL6/N mice using quantitative real-time PCR (5 or 20 ng RNA equivalent). n, number of biological replicates. Data were normalized to *B2m* and *Tfrc*. Statistics: one-way ANOVA followed by Bonferroni post-hoc test for whole-brain samples of different age and student´s t-test for brain regions; ***p < 0.001.

We also performed Western blot experiments (Fig. S3) to determine if Cav1.3 α1 subunits containing exons 8 plus 11 and 32 could contribute to the large and small size forms of Cav1.3 α1-immunoreactivity in the brain, previously found to express long and short C-termini [17]. Based on the observed molecular masses our findings are compatible with the interpretation that exons 11 and 32 can form part of native Cav1.3 channels in the brain (Fig. S3).

### Effects of alternative splicing of Cav1.3 α1-subunits in exons 8, 11 and 32 on channel gating

**Figure 3.**
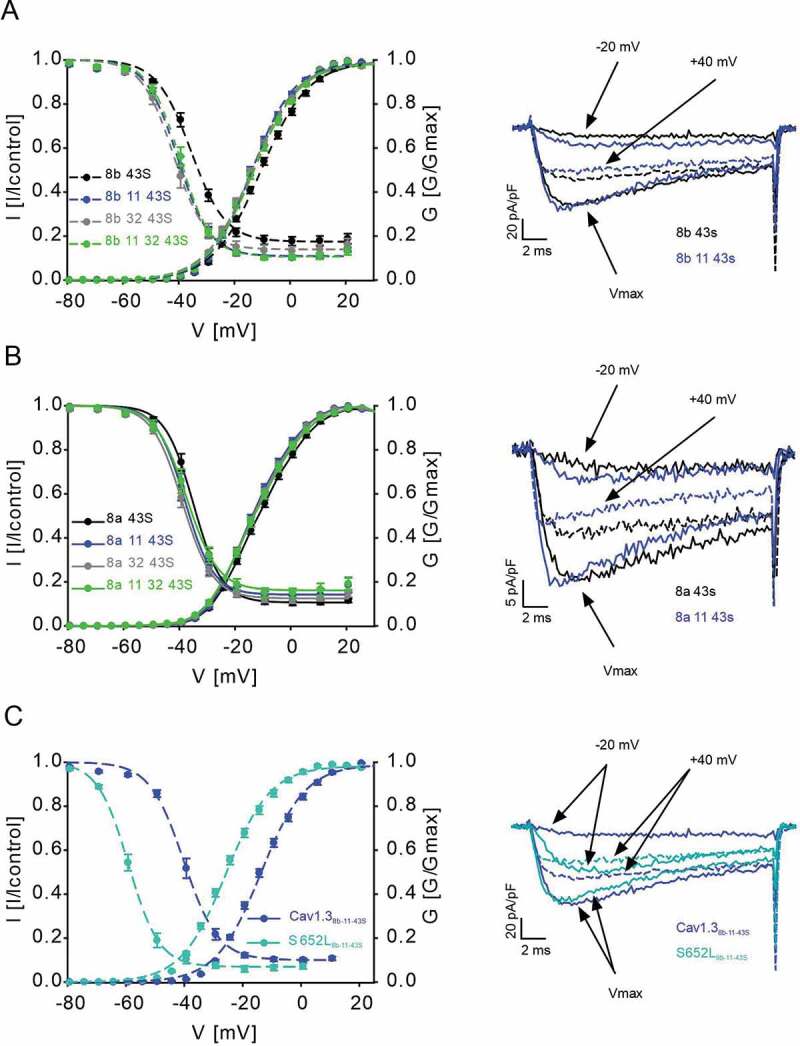
Effects of Cav1.3 splicing outside the C-terminus on channel gating

After having demonstrated the expression of these exons in different tissues, we functionally characterized splice variant combinations of Cav1.3_S_ containing either exon 8a or 8b with or without exons 11 and 32 or a combination of both (Cav1.3_8a/8b-43S_, Cav1.3_8a/8b-11-43S_, Cav1.3_8a/8b-32-43S_, Cav1.3_8a/8b-11-32-43S_) to test if they affect voltage-dependent channel gating as hypothesized. Introduction of exon 11 or 32 or both into Cav1.3_8b-43S_ significantly and reproducibly shifted V_0.5,act_ by -4 mV to more negative voltages (Figure 3a, for statistics, see Table 1). The voltage-dependence of inactivation (V_0.5,inact_) was also shifted to negative voltages by -4 to -6 mV. For exon 8a-containing channels these effects were smaller but also statistically significant for Cav1.3_8a-11-43S_ (V_0.5,act_) and Cav1.3_8a-32-43S_ (V_0.5,inact_; Figure 3b, Table 1). The fact that negative voltage shifts of both activation and inactivation gating were obtained in independent experiments with both exon 8 variants supports the robustness of our finding. Splicing caused no change of the inactivation time course during 5-s depolarizations to V_max_. (not shown). Despite smaller effects with exon 8a, our experiments were not designed to detect differences between exon 8a- and 8b-containing constructs.

**Table 1.** Activation and inactivation parameters of Cav1.3 8b and 8a splice variants

	Activation				Inactivation			
α1-subunit	V_0.5_ (mV)	Slope (mV)	V_rev_ (mV)	n	V_0.5_ (mV)	Slope (mV)	Non-inactivating (%)	n
8b 43S	-10.21± 0.62	8.51 ± 0.17	65.70 ± 0.88	25	-35.71 ± 0.83	5.52 ± 0.20	17.84 ± 1.98	22
8b 11 43S	-13.40± 0.63***	7.60 ± 0.15***	64.75 ± 0.72	29	-39.94 ± 0.97**	4.94 ± 0.19	10.82 ± 0.78**	21
8b 32 43S	-13.58 ± 0.55**	8.36 ± 0.15	63.21 ± 0.84	16	-41.53 ± 1.08***	5.21 ± 0.25	13.74 ± 2.23	12
8b 11 32 43S	-12.79 ± 0.58*	8.33 ± 0.12	65.23 ± 0.91	19	-39.54 ± 1.00*	4.90 ± 0.16	10.80 ± 1.19**	17
8a 43S	-10.99 ± 0.63	8.28 ± 0.16	65.67 ± 0.92	24	-34.79 ± 0.90	4.67 ± 0.19	10.65 ± 0.87	15
8a 11 43S	-13.21 ± 0.63*	7.6 ± 0.14**	63.75 ± 0.91	19	-37.99 ± 1.31	4.43 ± 0.18	14.23 ± 2.43	10
8a 32 43S	-13.03 ± 0.47	7.97 ± 0.13	63.80 ± 0.64	16	-38.82 ± 0.92*	5.04 ± 0.19	12.04 ± 1.28	13
8a 11 32 43S	-12.65 ± 0.76	8.23 ± 0.16	64.58 ± 0.81	17	-37.15 ± 1.36	4.91 ± 0.10	15.36 ± 2.14	13

Parameters were obtained from fitting normalized activation curves (G/G_max_) or normalized steady-state inactivation curves (I/I_control_). All values are presented as mean ± SEM and originate from >3 independent transfections. Statistics: one-way ANOVA followed by Bonferroni post-hoc test, *p < 0.05, **p < 0.01, ***p < 0.001 in comparison to 8b 43S or 8a 43S, respectively. n, number of experiments. V_0.5_, half-maximal activation/inactivation voltage; V_rev_, reversal potential.

Normalized steady-state activation and inactivation curves of Cav1.3_8b-43S_ (A) and Cav1.3_8a-43S_ (B) splice variants (left panels). All combinations shifted activation and inactivation to more hyperpolarized potentials compared to constructs lacking exons 11 and 32; for parameters and statistics see Table 1. C. Normalized activation and steady-state inactivation curves of Cav1.3_8b-11-43S_ vs S652L_8b-11-43S_ (left panel); for parameters and statistics see Table 2. The pronounced shift of activation and inactivation voltage dependence induced by disease variant S652L was maintained in exon 11-containing channels. A-C right panels: Representative I_Ca_ traces recorded during test potentials of -20 mV, V_max_ (solid lines) and +40 mV (dashed lines). Current amplitudes were normalized to the cell membrane capacitance (pA/pF). Currents <100 and >1000 pA were prospectively excluded from analysis. Data are presented as mean ± SEM and originate from >3 independent transfections for each construct.

Disease mutations also affect the voltage-dependence of activation and inactivation gating and previous experiments have employed channel variants lacking exons 8b, 11, and 32 [18–20,30]. We therefore also addressed the question whether their inclusion modifies mutational effects or whether the effects of these splice variants could be additive. For this purpose, we chose the previously reported mutation S652L (Figure 3c) which strongly shifts the voltage-dependence of gating to more negative potentials [20]. We tested the mutation in exon 11-containing construct Cav1.3_8b-11-43S_ as the reference, because exon 11 shifted activation parameters in both, exon 8a- and 8b-containing channels. Compared to Cav1.3_8b-11-43S_, mutant S652L_8b-11-43S_ caused a - 12.1 mV shift of V_0.5,act_ and a - 19.0 mV shift of V_0.5,inact_, (Figure 3c, Table 2) very similar in magnitude as shown recently [20] for S652L_S_ (V_0.5act_: -13.3 mV; V_0.5,inact_: -16.6 mV) (for statistics see Table 2).

**Table 2.** Activation and inactivation parameters of wildtype Cav1.3_8b-11-43S_ and S652L_8b-11-43S._

	Activation				Inactivation			
α1 subunit	V_0.5_ (mV)	Slope (mV)	V_rev_ (mV)	n	V_0.5_ (mV)	Slope (mV)	Non-inactivating (%)	n
Cav1.3_8b 11 43S_	-13.40 ± 0.63	7.60 ± 0.15	64.75 ± 0.72	29	-39.94 ± 0.97	4.94 ± 0.19	10.82 ± 0.78	21
S652L_8b-11-43S_	-25.50 ± 0.74***	7.61 ± 0.14	58.26 ± 0.71***	18	-58.92 ± 0.77***	4.93 ± 0.24	7.57 ± 1.37*	13

Parameters were obtained from fitting normalized activation curves (G/G_max_) or normalized steady-state inactivation curves (I/I_control_). All values are presented as mean ± SEM and originate from >3 independent transfections. Statistics: student´s t-test, *p < 0.05, ***p < 0.001. n, number of experiments. V_0.5_, half-maximal activation/inactivation voltage; V_rev_, reversal potential.

### *Effects of* CACNA1D *variant R498L in alternative exon 11*

**Figure 4.**
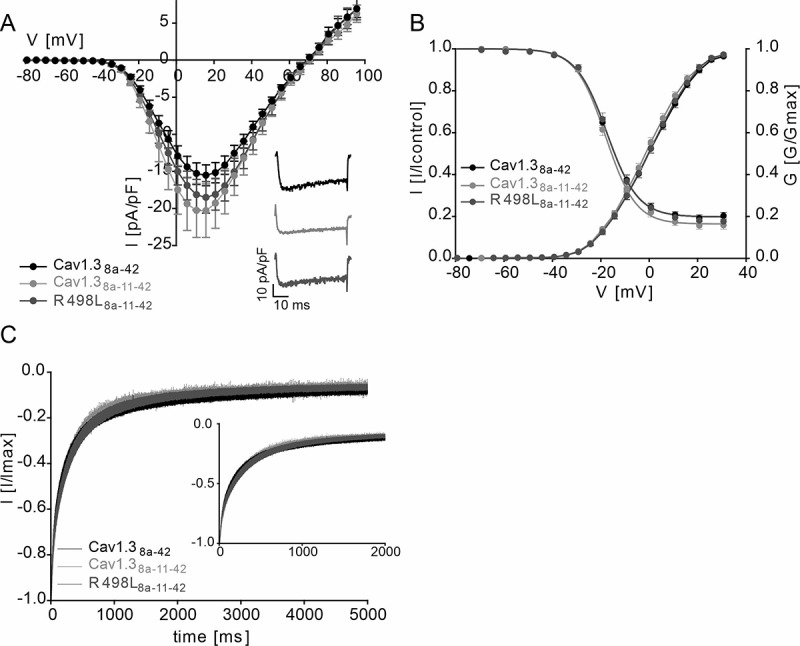
Effects of mutation R498L on channel gating

Since we found a regulatory role of cassette exon 11 on the voltage-dependent gating of Cav1.3 we further investigated if the *CACNA1D* variant R498L, residing in exon 11, also affects channel function. This variant has recently been identified by whole-exome sequencing in a patient with epilepsy [24] (see Supplemental Method section for details). However, genetic information from the patient’s parents was not available and hence its pathogenicity remained unclear. Demonstration of typical gating changes as observed for other pathogenic variants (see [23] for review) could therefore provide evidence for a role of this variant for the patient’s symptoms. To test this possibility, we employed the long Cav1.3 splice variant because it represents the reference construct previously used to study pathogenic *CACNA1D* variants. This also allowed us to test if introduction of exon 11 into the C-terminally long Cav1.3 _L_ isoform (termed Cav1.3_8a-42_ here for consistent nomenclature) affects gating properties. In contrast to Cav1.3_S_, exon 11 did not significantly shift the voltage-dependence of gating of Cav1.3_8a-11-42_ (Figure 4, Tables 3 and 4). R498L (R498L_8a-11-42_) induced no further change of the voltage-dependence of activation and inactivation gating and of the inactivation time course (Figure 4, Tables 3 and 4).

**Table 3.** Activation and inactivation parameters of mutations R498L

	Activation				Inactivation			
α1 subunit	V_0.5_ (mV)	Slope (mV)	V_rev_ (mV)	n	V_0.5_ (mV)	Slope (mV)	Non-inactivating (%)	n
Cav1.3_8a-42_	0.45 ± 0.90	9.51 ± 0.15	66.56 ± 0.86	44	-17.35 ± 0.76	6.02 ± 0.18	19.56 ± 1.25	33
Cav1.3_8a-11-42_	-1.31 ± 0.88	9.32 ± 0.20	63.75 ± 0.97	25	-17.63 ± 0.81	6.01 ± 0.30	15.94 ± 1.76	17
R498L_8a-11-42_	0.04 ± 1.03	9.28 ± 0.11	66.14 ± 1.04	30	-16.48 ± 0.88	6.03 ± 0.37	17.79 ± 1.62	23

Parameters were obtained from fitting normalized activation curves (G/G_max_) or normalized steady-state inactivation curves (I/I_control_). All values are presented as mean ± SEM and originate from >3 independent transfections. Statistics: One-way ANOVA, followed by Bonferroni multiple comparison posttest. n, number of experiments. V_0.5_, half-maximal activation/inactivation voltage; V_rev_, reversal potential.

**Table 4.** Normalized inactivation kinetic parameters of mutation R498L

	Remaining I_Ca_ [%]							
α1 subunit	r_50_	r_100_	r_250_	r_500_	r_1000_	r_5000_	n	
Cav1.3_8a-42_	69.41 ± 2.54	57.47 ± 2.65	39.14 ± 2.35	26.48 ± 1.94	17.72 ± 1.56	8.05 ± 0.94	28	
Cav1.3_8a-11-42_	72.97 ± 3.29	61.20 ± 3.12	40.67 ± 2.69	25.87 ± 2.13	15.61 ± 1.80	6.47 ± 1.25	13	
R498L_8a-11-42_	70.66 ± 2.88	60.22 ± 3.00	42.17 ± 2.93	27.64 ± 2.46	16.67 ± 1.72	6.90 ± 0.84	22	

r-values represent the fraction of remaining I_Ca_ after 50, 100, 250, 500, 1000 and 5000 ms upon a 5 s depolarization to the voltage of maximal inward current (V_max_). All values are presented as mean ± SEM and originate from >3 independent transfections. Statistics: one-way ANOVA followed by Bonferroni multiple comparison post-test R498L_8a-11-42_ in comparison to Cav1.3_8a-42_ and Cav1.3_8a-11-42_; n, number of experiments.

A. Current-voltage relationships (I_Ca_; mean ± SEM) of Cav1.3_8a-42_ and Cav1.3_8a-11-42_ vs R498L_8a-11-42_ recorded in parallel on the same day. Inset: Representative I_Ca_ traces of Cav1.3_8a-42_, Cav1.3_8a-11-42_ and R498L_8a-11-42_ upon depolarization to the V_max_. B. Normalized steady-state activation and inactivation curves of Cav1.3_8a-42_ and Cav1.3_8a-11-42_ vs R498L_8a-11-42_. Data are presented as mean ± SEM; for parameters and statistics see Table 3. C. Inactivation kinetics of Cav1.3_8a-42_ and Cav1.3_8a-11-42_ vs R498L_8a-11-42_ during a 5-s depolarization to V_max_ with Ca^2+^ as a charge carrier showing no difference in inactivation kinetics between Cav1.3_8a-42_ and Cav1.3_8a-11-42_ vs R498L_8a-11-42_. Inset shows the first 2000 ms. Data are presented as mean ± SEM; for statistics see Table 4. Data were collected from >3 independent transfections.

Discussion

In contrast to Cav1.2 [33], only a small portion of Cav1.3 splice variants has yet been identified and understood in terms of their roles for channel function. C-terminal splice variants have been previously characterized, which differ in their voltage-dependence of activation, Ca^2+^-dependent inactivation properties, and dihydropyridine sensitivity [15,16,34], while the effects of splicing upstream of the C-terminus are largely unexplored. Here we provide experimental evidence for a critical role of alternative splicing in exons 11 and 32 for stabilizing negative gating properties of Cav1.3 channels thus shifting its operating range even more toward subthreshold voltages. This effect appears to be stronger in exon 8b-containing short variants. In Cav1.3_S_, the effects of exon 11 and 32 are not additive, suggesting that they either act through stabilizing similar conformational changes or dominantly via different mechanisms.

Alternative splicing at a homologous position in exon 32 of Cav1.1 channels, i.e. developmentally regulated insertion of exon 29 right-shifts voltage-dependence of activation by ≈30 mV [35]. This regulation depends on interactions between positive gating charges and a negative countercharge in the voltage-sensing domain of Ca^V^1.1^35^. Neutralization of the corresponding negative countercharge in Cav1.3 lacking exon 32 and exon 11 (Cav1.3_8a-42_) also shifts V_0.5,act_ to more positive voltages (although only slightly, ≈ 5 mV, ^35^). It therefore needs to be tested if insertion of exon 32 into Cav1.3_S_ also affects interactions between positive gating charges and negative countercharges within its voltage-sensing domain that could explain the observed gating changes described here. In contrast, it is less clear how exon 11 could affect gating. Some clues come from the fact that it is positioned in the I-II-linker between two important regulatory domains, which are both coupled to voltage-dependent gating (cf. Figure 1a): the auxiliary β-subunit interaction domain is located upstream of exon 11 and a regulatory cluster of positive residues forms an amphiphilic helix interacting with the cytoplasmic leaflet of membrane lipids at the C-terminal end of the I-II-loop [36]. However, in the absence of high-resolution structures of this region [37], it remains unclear if exon 11 splicing acts through these regulatory domains.

Previous biophysical characterizations of the role of exons 11 and 32 were not conclusive suggesting no gating changes by both exons when expressed in Xenopus oocytes [21], no changes for exon 11 when expressed in tsA-201 cells [38] (both measured in 5 mM Ba^2+^) or a modest depolarizing shift in V_0.5,act_ of +3 mV (i.e. the opposite effect as reported here, measured in 10 mM Ba^2+^) upon inclusion of exon 32 [22]. Exclusion of the corresponding exon (exon 33) in Cav1.2 α1-subunits resulted in a hyperpolarizing shift in activation of about 5–10 mV [35,39], which has been associated with the development of ventricular arrhythmia and a higher risk for heart failure [39].

We show convincingly that exons 11 and 32 introduce negative shifts in the voltage-dependence of activation and inactivation in Cav1.3_S_. These changes appear small but it should be kept in mind that even minor gating changes of Ca^2+^-channels can affect neuronal excitability in a relevant manner. This is also evident from Cav2.1 (*CACNA1A*) gain-of-function mutations associated with familial hemiplegic migraine type-1. Here, only an about 5 mV shift of current activation to more negative potentials in heterozygous Cav2.1^S218L^ knock-in mice [40] was sufficient to significantly enhance synaptic transmission at the neuromuscular junction and to lower the threshold for initiation and the propagation velocity of cortical spreading depression, which is a key player in the pathogenesis of migraine [41,42].

Negative shifts in the voltage-dependence of activation have previously been identified as one of the disease-causing mechanism of *de novo CACNA1D* missense mutations in individuals with a broad neurodevelopmental disease spectrum [18]. Based on our knowledge that C-terminal splicing differentially affects Cav1.3 channel gating, we routinely characterize such *CACNA1D* mutations in long and in short splice variants, which are both abundantly expressed in the brain [15,34]. Although small differences exist, we found mutation-induced gating pathology both in long and in short C-terminal splice variants [18,20,30]. Here we show that pathogenic gating changes induced by mutation S652L are fully preserved and essentially additive to the negative shift induced by exon 11. In this way, mutation S652L as well as other mutations that shift voltage-dependence of activation to more negative potentials may elicit differential effects in various brain regions depending on the exon composition of the channels. In a neuron of a S652L heterozygous individual alternative splicing within and outside the C-terminus could therefore support a > -25 mV difference in V_0.5,act_ and a > -30 mV difference in V_0.5,inact_ between C-terminally long wild-type (V_0.5,act_ = -0.18 mV, V_0.5,inact_ = -25.7 mV [43]) and short S652L-mutated Cav1.3 channels (Table 2).

We did not identify gating changes for *CACNA1D* variant R498L, which is located in exon 11. Although we cannot exclude that this variant affects channel function through a mechanism not detectable by heterologous expression, our data do not allow to classify this variant as a pathogenic one.

Only few studies systematically analyzed alternative exon 11 and exon 32 splicing in Cav1.3 ɑ1-subunits, which both can be included or skipped and are located in the I–II loop and in the extracellular IVS3-4 linker, respectively. For instance, inclusion of cassette exons 11 and 32 has been identified in human and rat brain and in pancreatic tissue [44–47]. In a recent unpublished report, LaCarubba and colleagues systematically also analyzed the expression of exons 11 and 32 in mouse whole brains at different postnatal ages as well as in fetal and adult human whole brains. They could show that both are upregulated during development and are therefore dominant variants in adult mouse and human brains [38]. Here we could confirm the expression of exons 11 and 32 not only in the brain but also in MCCs and in the cochlea. In addition, we provide evidence that exons 11 and 32 can be present within a single transcript in the brain. Our Western blot analysis also provide indirect evidence that exons 11 and 32 are part of native α1 subunits in mouse brain.

In this study, we also focused on the splicing of exons 8a/8b, 11 and 32. Exons 8a and 8b are mutually exclusive, located in the first domain in segment 6 (IS6), which forms part of the activation gate of the pore and are essential for channel function. Previous reports revealed a higher expression of exon 8b in the sinoatrial node and cochlear hairs cells, which explains why a Cav1.3 loss-of-function mutation in exon 8b results in bradycardia and congenital deafness (SANDD; OMIM #614,896 [10,48]). Here we show a much higher expression of exon 8b in most brain areas. Therefore, a large reduction of functional Cav1.3 channels must also have occurred in the CNS of these SANDD patients. Interestingly, some brain regions express a higher relative amount of exon 8a-containing channels, including areas with dopamine neurons (SN, VTA) and the cerebellum. However, based on our data it appears that splicing in this position does not cause major effects on channel gating. Whether exon 8 splicing affects single-channel conductance has yet to be determined. At present, the physiological role of exon 8 splicing remains unknown.

Taken together our results encourage further studies of the role of alternative splicing for the regulation of Cav1.3 channels. In particular, based on the available high-resolution structure of the IVS3-S4 linker region molecular modeling should aid in generating a testable hypothesis about how exon 32 can affect channel gating. Moreover, novel Cav1.3 splice-variants identified by long-read sequencing as recently described for Cav1.2 [33] may even further expand the functional diversity of Cav1.3 channels [49,50].

## Supplementary Material

Supplemental Material

Supplemental Material

## Data Availability

The data that support the findings of this study are available from the corresponding author, upon reasonable request.
