# Supplementary figures and images for: Stabilization of negative activation voltages of Cav1.3 L-Type Ca2+-channels by alternative splicing
Source: Channels (Austin). 2020 Dec 31;15(1):38–52. doi: 10.1080/19336950.2020.1859260 (PMC7781618; doi:10.1080/19336950.2020.1859260)

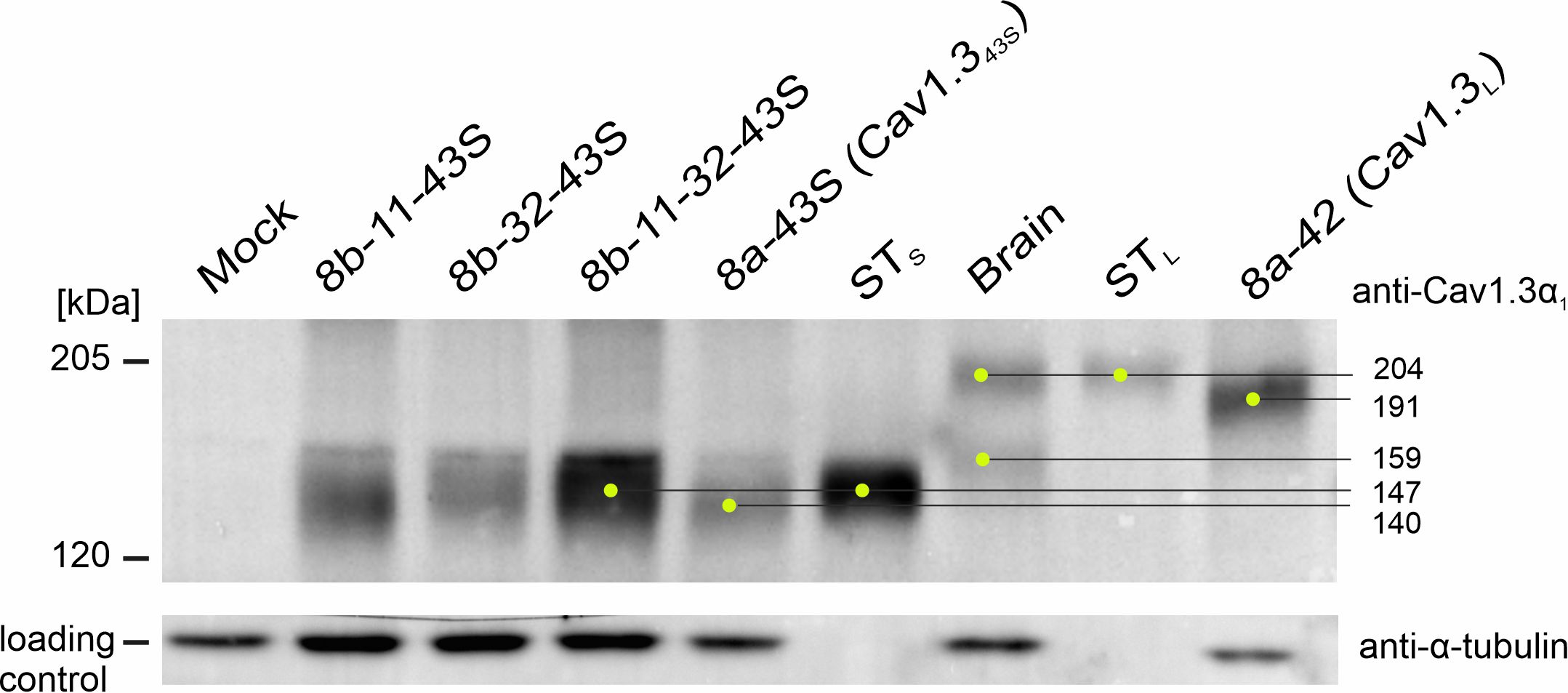

Supplement: Supplemental Material [file KCHL_A_1859260_SM4513.jpg]

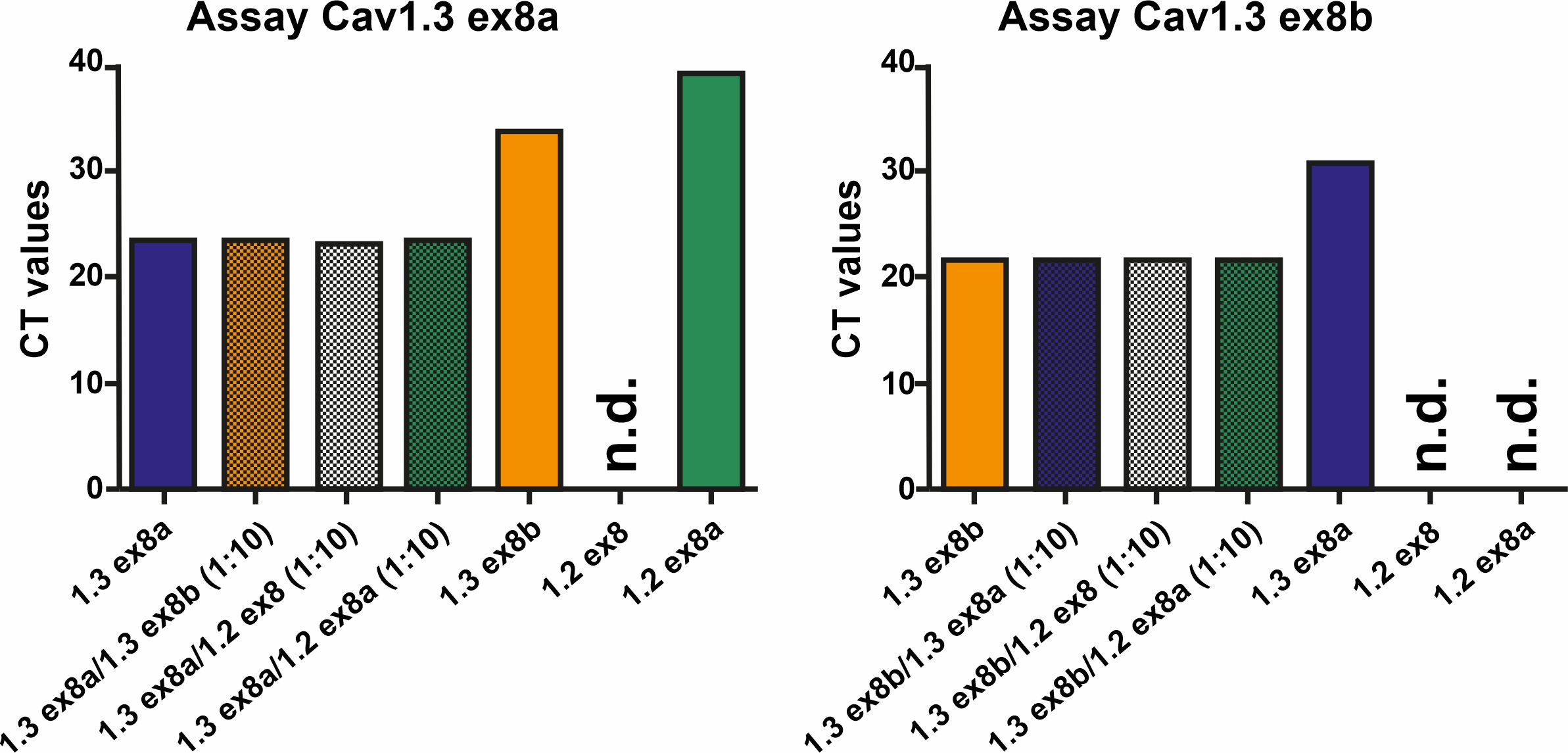

Supplement: Supplemental Material [file KCHL_A_1859260_SM4510.jpg]
